# Wireless Sensor Networks Composed of Standard Microcomputers and Smartphones for Applications in Structural Health Monitoring †

**DOI:** 10.3390/s19092070

**Published:** 2019-05-03

**Authors:** Guido Morgenthal, Jan Frederick Eick, Sebastian Rau, Jakob Taraben

**Affiliations:** Chair of Modelling and Simulation of Structures, Bauhaus University Weimar, 99421 Weimar, Germany; jan-frederick.eick@uni-weimar.de (J.F.E.); sebastian.rau@uni-weimar.de (S.R.); jakob.taraben@uni-weimar.de (J.T.)

**Keywords:** Raspberry Pi, smartphones, wireless sensor networks, vibration measurements

## Abstract

Wireless sensor networks have attracted great attention for applications in structural health monitoring due to their ease of use, flexibility of deployment, and cost-effectiveness. This paper presents a software framework for WiFi-based wireless sensor networks composed of low-cost mass market single-board computers. A number of specific system-level software components were developed to enable robust data acquisition, data processing, sensor network communication, and timing with a focus on structural health monitoring (SHM) applications. The framework was validated on Raspberry Pi computers, and its performance was studied in detail. The paper presents several characteristics of the measurement quality such as sampling accuracy and time synchronization and discusses the specific limitations of the system. The implementation includes a complementary smartphone application that is utilized for data acquisition, visualization, and analysis. A prototypical implementation further demonstrates the feasibility of integrating smartphones as data acquisition nodes into the network, utilizing their internal sensors. The measurement system was employed in several monitoring campaigns, three of which are documented in detail. The suitability of the system is evaluated based on comparisons of target quantities with reference measurements. The results indicate that the presented system can robustly achieve a measurement performance commensurate with that required in many typical SHM tasks such as modal identification. As such, it represents a cost-effective alternative to more traditional monitoring solutions.

## 1. Introduction

The mechanical properties of structures change over the course of their lifetime. Changes related to deterioration such as damage may be safety-relevant and need to be detected. Modern sensor-based methods to determine the behavior of the structure and to assess its condition are the subject of structural health monitoring (SHM). Typical SHM processes implement a damage detection strategy that involves the monitoring of a structure over a period of time with a suitably selected measurement system and identifying damage indicators or damage-sensitive features. A common non-destructive approach to monitor damage is through vibration-based measurements using acceleration sensors. Signal processing and system identification techniques can be used to compute global properties such as natural frequencies and structural mode shapes from the transient data, and model-based calibration methods allow for the detection of changes in mechanical characteristics from changes in these. The quality of the identified structural properties and as such the capability of detecting system changes such as damage are directly associated with the characteristics of the measurement system.

Compared with traditional wire-based strategies for monitoring civil structures, wireless mobile mesh network systems (wireless sensor networks (WSNs)) have proven themselves as a time- and cost-efficient solution, as they aim for a faster and more adaptive examination process. Many studies are published that focus on the development of WSNs including the aspect of reliable data transport, the compression of data, data synchronization, and distributed computing [1,2] as well as their application to different types of structures such as bridges and towers [3,4]. However, the different WSNs developed [4] are not designed for mobile usage, which would also allow flexible integration of different types of sensor nodes. In contrast to permanent installations of sensor networks on structures, there is a growing need for flexible and mobile hardware solutions. This demand can be addressed by the use of microcomputer hardware. Considering the SHM context, only a few studies are focused on measuring acceleration by only using microcomputer and external microelectromechanical system (MEMS)-based sensors. So far, mass market microcomputers and microcontrollers have been used as control platforms, which are accessed via WiFi from a computer, and Raspberry Pi (RPi) microcomputers have been connected to ADXL345 sensors [5] and directly to MPU-6050 accelerometers [6] or via Arduino Mega microcontroller [7]. Besides the application for vibration measurements, other wireless monitoring systems employing cost-effective microcomputers and microcontrollers in combination with specific piezoelectric sensors (PZTs) have been developed to identify local damage in reinforced concrete elements. These systems are based on the electromechanical impedance (EMI) concept and integrate Raspberry Pi [8] and ATmega microcontrollers [9].

Mobile measurement systems, i.e., smartphone and tablet devices, have been applied to monitor infrastructures, such as bridges. Internal MEMS-based accelerometers are typically utilized to record structural vibrations in order to extract dynamic properties. Sensing properties vary drastically between the available smartphones since different software systems and hardware components are integrated. Therefore, different devices will offer different measurement quality, and the imposed limitations can solely be expressed as smartphone-dependent. Characteristics such as the maximum sampling rate, the range, and the resolution have been investigated in various studies [6,10,11,12], where maximum sampling rates of 225 Hz and resolvable accelerations down to 1 mm/s${}^{2}$ were found. However, the latest smartphone models are not covered. In these studies, the cable-force estimation of stay cables [13] or suspension bridge hangers [14] of long-span bridges has been a major application. The same method was also used to determine the prestressing forces of external post-tensioning tendons at a highway bridge [15]. Mechanical vibrations of stay cables were also investigated by utilizing the integrated camera of the smartphone, whereby the natural frequencies were evaluated from stationary as well as handheld smartphone cameras by tracking the displacement of the cables [16]. Since the operating system can influence the achievable measurement quality, e.g., depending on battery status or the number of processes running in parallel, external sensors have also been used for data collection. The connection between smartphone and external sensors has been established wired [13] or wirelessly via microcontroller [6]. In order to collect, store, process, and visualize acceleration data, smartphone applications have been developed for Android [6,10] as well as for iOS [13,14] operating systems, which allow data handling within a single device.

Smartphone-based vibration measurements were applied in [17] for the identification of dynamic properties on rural bridges. The application of smartphones for damage detection was studied in [18]. There, a small-scale steel frame model was subjected to base excitation, and peak acceleration data obtained from an internal sensor was in good agreement with the one determined from traditional piezoelectric sensors, though greater variance was found for the displacements.

The enormous increase in applications of sensor-based technology in the scope of SHM demonstrates the necessity of improved, high-performance, mobile technologies. With the rapid development and popularization of smartphones, the adoption of these became increasingly interesting to the SHM community. The presented work concentrates on the properties and advantages of the software framework designed for single-board computers to measure vibrations via sensors attached to microcontrollers or nested in mobile devices. Such modern devices include smartphones and tablets equipped with considerable computational resources and large memory capacity. Unlike traditional monitoring systems, these widely available devices combine the recording, storage, processing, transfer, and visualization of data within one unit. Due to vast differences in measurement properties arising from the various built-in sensors on the market and their qualities, external, low-cost sensors connected to RPi are included complementary to the mentioned hardware components for additional data acquisition. All aforementioned systems utilize the MEMS–based acceleration sensors to determine the system properties of structures by measuring structural vibrations. As past studies have already shown, the sensor resolution and sampling rate, which result from differences in accelerometers and sensor driver software configuration, affect the properties of measurement data directly [6]. Based on this fact, sensor characteristics are further investigated with respect to the hardware used. To demonstrate the performance of the technology platform, two types of application areas are showcased. Preliminary work included an evaluation of measurements on stay-cables during the construction of Queensferry Crossing [6], where tension forces were determined and compared with direct force measurements. Vibration measurements on bridge piers were performed on the Schindgraben bridge during blasting activities to determine vibration velocities.

The technology platform presented here employs standard mass market hardware and features a software framework specifically geared towards optimum utilization of system resources in the context of WiFi-based meshed sensor network transient measurements. In contrast to existing methods [19], the approach presented in this paper deliberately refrains from using additional microcontroller hardware to facilitate data acquisition from sensor hardware. A primary design goal was the implementation in Python as a high level programming language to provide transparency and facilitate future extensions. Further, the systems can be readily used in education where students are enabled and encouraged to comprehend and extend every stage of the data processing pipeline. Time-critical sections have been profiled to identify bottlenecks of performance and carefully optimized to meet deterministic run-time guarantees as well as possible. The authors make the software framework available alongside this publication under the name *RasPyre* [20] which can be found on the website of the authors.

## 2. Hardware and Measurement System

### 2.1. Concept

Since conventional measurement systems in the context of SHM are costly and often bound to specific hardware, the main goal of the present work is to provide two alternatives based on consumer grade and mobile hardware. The following two approaches have been previously presented in [21].

The first approach uses off-the-shelf single-board computers (Raspberry Pi 3 Model B+), which are equipped with an interface board developed by the authors that provides connections for different digital sensor interfaces and analog-to-digital converters (ADC) for connecting analog sensors, as well as a battery-buffered realtime clock. Note however, that the bare RPi can also be used alone as long as the standard GPIO connectors can accommodate the sensor setup. The RPi hardware setup is used as a node within an established wireless mesh network that is dynamically extendable with additional nodes during runtime. Each node has the ability to function as sensor node collecting data employing the attached sensors and to act as an access point (AP) simultaneously. Configured network ports serve as a communication channel where all commands and requests are transmitted from a central node to each available WSN node. Due to the automatic synchronization of the sensor nodes prior to measurements, simultaneous measurements are also possible.

The software framework is designed to be platform-independent, so hardware systems other than the RPi are applicable. The only platform requirements are that a Linux operating system is used and that WiFi is available for automatized meshing. At the time of writing, the presented implementation, however, has only been extensively tested on the RPi platform.

The second approach comprises an Android application (App) customized for vibration measurements using the built-in accelerometer of smartphones and tablets. In addition to the data collection, the developed application features several tools for data processing, allowing interactive signal analysis. Thus, the app enables the user to perform further calculations, e.g., the determination of tendon forces or the integration of accelerations to determine velocity and displacement time histories. Besides the measurement data, the application can store a few different types of information about the conducted measurement or inspection such as photos, notes, and sketches directly on the device. This second approach does not include the meshing functions like the RPi system does. Therefore, the smartphone in connection to the app developed is not able to be used as a network node without further changes to the system architecture.

In order to exploit the full potential of the showcased software framework, the feasibility of the combination of both approaches is investigated. The authors present a way to utilize smartphones as user facing control units as well as regular sensor nodes in the WSN.

### 2.2. Single-Board Computer Platform

**Figure 1:**
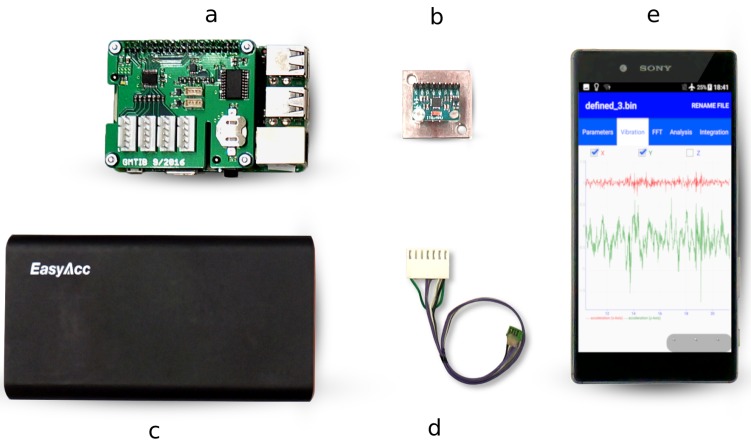
Hardware components of the measurement system. (**a**) The RPi with an additional sensor board; (**b**) the external sensor MPU-6050; (**c**) the battery power supply; (**d**) the wire to connect the RPi and the external sensor; (**e**) the smartphone Sony Xperia Z5 running control and analysis app.

The RPi 3 Model B+ was used as a measurement hardware platform to utilize external high quality sensors. A single hardware node is comprised of the RPi single-board computer itself (a), one or several external sensors (b), a battery supply (c), the specific sensor connection cable (d), and an Android smartphone with the control app (e), as shown in Figure 1. Different types of sensors can be integrated in the measurement system ranging from low-cost sensors with an inter-integrated circuit (I${}^{2}$C) interface over sensors connected via serial peripheral interface (SPI) to analog sensors requiring an ADC. The above-mentioned custom-design interface boards allow for the connection of several I${}^{2}$C- and SPI-based digital sensors as well as feature ADCs to connect analog sensors. A self-designed interface board expands the existing 2-Pin-I${}^{2}$C-port such that four sensors can be connected. In the same manner, an incorporated demultiplexer expands the two provided SPI chip select ports of the RPi to allow for the attachment of several SPI devices.

The RPi is equipped with a 2.4 GHz WiFi 802.11n Broadcom FullMAC chip, which is utilized by the developed software framework to provide a wireless mesh network.

**Figure 2:**
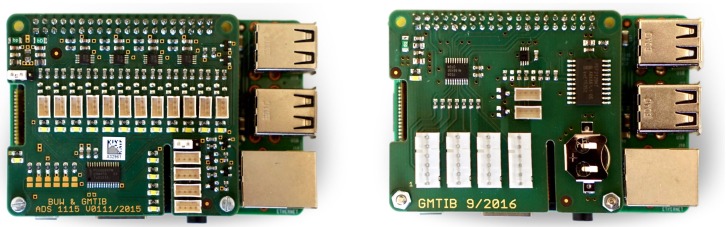
Additional sensor boards. (**Left**) A sensor board equipped with four ADS1115 16 bit ADCs [22], port headers to connect up to 12 channels of analog sensors, and four additional ports for the I${}^{2}$C-connected sensor hardware. (**Right**) A sensor board equipped with a battery-backed CMOS real time clock [23], four port headers to connect SPI-based sensors, and two additional ports for the I${}^{2}$C-connected sensor hardware.

To faciliate the easy and quick connection of various sensor hardware, the authors developed two sensor boards that can be connected on top of the RPi’s GPIO pin header. Figure 2 (left) shows the connector board equipped with four programmable ADS1115 ADCs [22] to connect three input channels of analog sensor hardware for each. Figure 2 (right) shows the connector board for primary use with the digital sensor hardware. The board features a battery-backed PCF2129 CMOS real time clock [23] to keep system time when the RPi is disconnected from a power source as well as four Molex six-way headers to connect the SPI-based sensor hardware. Both sensor boards are equipped with two, or respectively four JST four-way headers connected to the I${}^{2}$C bus of the RPi.

It should be noted that the software framework presented in this paper does not depend on any additional hardware. Individual sensor hardware can be connected to the RPi directly to the specific pins of the GPIO header.

To facilitate RPi-based measurements, a corresponding software framework was developed. Each node uses a modified version of the Debian-based computer operating system Raspbian including a customized version of the Linux kernel to enable the execution of real-time tasks on the platform [24]. This modification is key to facilitating high-frequency sensor data acquisition without the need of an additional dedicated microcontroller.

The actual software framework is implemented in the Python programming language to accelerate the software development cycle. It is comprised of three core parts:

- a unified software interface communicating with the attached sensor hardware,
- a data acquisition and transport process, and
- a remote procedure call (RPC) server to expose a control interface to the smartphone application.

When a measurement task is initiated, the RPC server spawns a carefully designed process that handles the actual polling of the sensor hardware. The process utilizes a real-time scheduling class of the operating system with a high priority to ensure low latencies during the execution of the periodic sampling task. Furthermore, the operating system is instructed to associate all current and future memory allocations to physical memory and prevent these memory pages from being paged-out at any time, even when the system is facing memory pressure. A shared memory circular buffer is used to transport the acquired sensor data to a non-time-critical handler process, which is used to store the data on the local file system or to stream it over the network. This approach allows stable sensor polling with a frequency of more than 1 kHz. The handler process can be configured to perform initial pre-processing, such as smoothing or downsampling of the sensor data before it is further processed.

The hardware facing software component is designed as a plugin mechanism to facilitate the rapid development of a variety of optimized sensor drivers. A driver module can be independently deployed and loaded during run-time depending on the attached sensor hardware and the task at hand. Currently, driver modules that have been created for the sensor hardware are listed in Table 1.

### 2.3. Smartphone Platform

**Figure 3:**
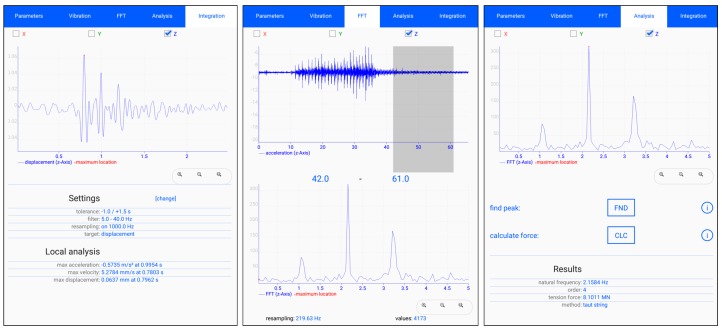
Screenshots of the user interface of the Android app. (**Left**) Visualization and settings dialog for the integrated signal of a captured blast impact operation; (**middle**) selection dialog of the signal range; (**right**) dialog for the semi-automated peak selection and tension force calculation.

An Android application was developed by the authors in a previous work to assist the process of smartphone-based measurements [6]. The app offers a broad set of tooling options and features a user-friendly interface as shown in Figure 3. The app provides several options for measurements using the on-board accelerometer of the smartphone. Besides general settings (e.g., the sampling rate, the measurement duration, or the sensing axes), there are three different ways to trigger and store the recording of an acceleration signal:

- a simple start-stop-measurement where the length of the signal is defined by the user activating and deactivating the measurement by hand,
- a buffered-measurement that runs continuously and stores only the data of a time-limited buffer into a file when deactivated by hand, and
- an event-triggered-measurement that runs continuously and stores only the acceleration data into a file when a defined signal threshold has been exceeded (e.g., during earthquake or explosions).

Additionally, a delayed measurement after a defined time or for a simultaneous start on the next clock minute, as applied in [17], is possible.

Data are stored temporarily in the working memory during data acquisition to avoid delays. After a measurement is completed, the data are saved to the internal storage of the smartphone within a defined tree structure of folders, and this structure is used as a database to manage projects and sub-elements. Each of the sub-elements in the folder structure holds a list of assigned measurements and enables the user to additionally create and store images, videos, voice memos, notes, sketches, location coordinates, or specific properties of building elements. This facilitates a comprehensive documentation of each measurement task and its environment at the investigated building element for the evaluation of analysis results or a detailed survey report.

Furthermore, there are various in-app signal analysis tools to evaluate the measured data directly on the smartphone. As an overview, a list of meta data is attached to the measurement file, and the actual signal is visualized by a plot view. Another function applies a fast Fourier transform (FFT) on a section of the signal defined in an interactive plot view that enables one to cut off unnecessary or externally disturbed parts of the signal. Once the result of the FFT is computed, the frequency domain output can be used as input for a further option to calculate the tension force in structural elements such as bridge cables and external tendons. The taut string approach is used with parameters for stiffness and mass combined with an eigenfrequency obtained from a semi-automated peak-picking routine. An additional function provides the integration of the acceleration signal to the corresponding vibration velocity and displacement in the time domain applying an optional frequency filter for signal corrections.

Connecting the smartphone to an RPi to receive the measurement data from the attached sensors as previously explained enables the integration and analysis of RPi data on the smartphone as well.

### 2.4. Mesh Network

A mesh network is used to build a flexible mobile ad hoc sensor network on site, which enables the user to reach every node in the mesh network by connecting to a dedicated node of choice. Furthermore, this approach allows for reaction to changing requirements prior and during measurement campaigns. The network quality between hard-to-reach measurement locations can be improved by strategically placing additional RPi nodes to act as a WiFi range extender with or without sensing functionality.

The operating system is configured to generate a unique hostname and network IP address during system boot, using the unique MAC address of its on-board WiFi interface as a seed. Subsequently, the WiFi interface is configured to join a mesh network that is implemented using the open link state routing (OLSR) protocol [25]. The OLSR service floods neighboring nodes in regular intervals with routing information packets to discover the topology of the sensor network. All nodes keep the state of the topology as local entries in their own routing table. Unfortunately, to date it is not possible to connect to the mesh network directly with an unmodified Android smartphone due to the lack of ad hoc network modes. To bypass this limitation, the authors designed a mechanism to declare an arbitrary node as a portal to the mesh network by plugging in a separate WiFi-USB adapter. The device management subsystem detects the additional network adapter and spawns a local wireless AP, enabling nearby smartphones and computers to connect and reach every node in the mesh network.

At the time of writing, very few Android devices feature WiFi chipsets that support ad hoc mode. Hence, a direct integration of smartphones into the mesh network is not feasible. The authors developed a method to bypass this restriction. To integrate a single Android device into the mesh, the user first connects the device manually to a nearby portal node. Each RPi node generates the IP address of the mesh interface from the least significant three octets of the MAC address of the onboard WiFi interface. For the MAC address B8:27:EB:AA:BB:CC, the mesh interface is configured to the IPv4 address 10.170.187.204. The additional USB WiFi dongle designates the node to a portal node. The systems AP daemon is then configured to assign connecting clients an IP address via the dynamic host configuration protocol (DHCP) in a class C subnet of the first three octets of the IP address of the mesh interface. e.g., given the mesh interface IP address 10.170.187.204, the AP daemon will be automatically set up using the address 10.170.187.1 and will lease IP addresses in the 10.170.187.0/24 subnet, excluding the address which is already assigned to the mesh interface. Next, the OLSR daemon on the node then registers the AP subnet as a home network and publishes this information using host and network association (HNA) packets to the remaining nodes. After successful connection to a portal node, the smartphone then starts to broadcasts its IP address and its hostname using the multicast DNS (mDNS) protocol [26]. Analogously to the RPi software framework, an RPC server is then spawned, which provides a subset of the interface of the RPi platform. The user is then able to utilize the smartphone like any other node in the mesh network and trigger measurements as well as transfer accelerometer data in real time.

**Figure 4:**
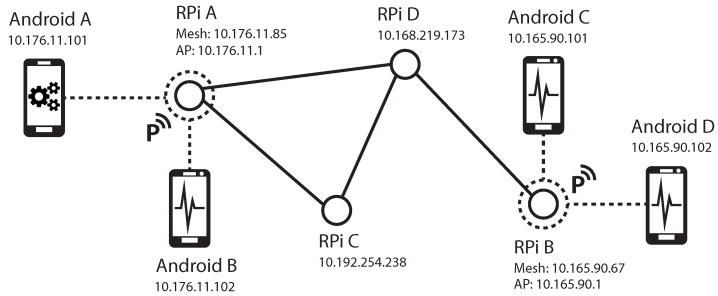
Exemplary network topology consisting of four RPi nodes and four smartphones.

Figure 4 illustrates an exemplary network topology consisting of four RPi nodes and four smartphones, three of which are configured as sensor nodes. The RPi nodes automatically configured their mesh interfaces and joined the mesh with the ESSID “raspyre.olsr.” RPi A and RPi B are also equipped with an additional WiFi-adapter and are automatically configured as portal nodes with their corresponding AP gateway addresses. Two smartphone devices Android C and Android D are connected to the portal node RPi C, while the two smartphone devices Android A and Android B are connected to the node RPi A. Each node announces its own IP address and hostname, which are discovered by the client app running on Android A.

### 2.5. Interfaces of Measurement System

An RPC server component is utilized to listen for commands to configure attached sensor hardware, to discover nodes in the mesh network and to control actual measurements. Moreover, rudimentary support to synchronize the system time between nodes is provided using the network time protocol (NTP). Users can select an arbitrary node in the network as the reference time server to which every other node synchronizes its clock. This procedure can be performed prior to measurements to guarantee coherence between time stamps of measurements that are carried out by different sensor nodes at the same time.

**Figure 5:**
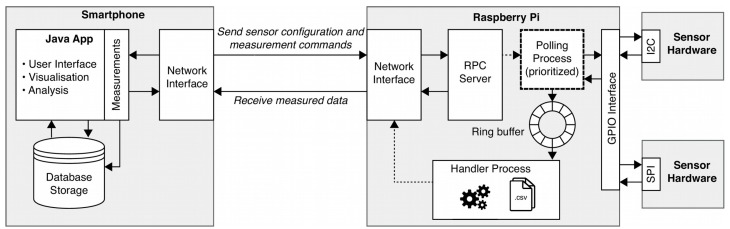
Schematic of signal processing and communication between smartphone and one exemplary RPi node in the mesh network.

To allow for efficient data handling and administration, several interfaces are provided by the smartphone application. A schematic of the wireless network connection to the wireless sensor mesh network with external sensors, which is realized by an XMLRPC protocol is shown in Figure 5. For the transfer of stored sensor data on the nodes, the common hypertext transfer protocol (HTTP) is used.

A measurement using the RPi requires the two devices to establish a network connection. To this end, the dedicated node to establish a portal to the mesh network opens a wireless access point to which the smartphone connects as a client. Within the app, the user is able to discover all nodes of the mesh network that are reachable via this portal node. In order to take a measurement, the desired nodes have to be chosen, which will receive a configuration command with the defined measurement settings for the sensor interface framework to trigger a measurement. The user can define whether the nodes store the measurement data locally or they directly transfer the data to the mobile device. After the completion of a measurement, the app can download the stored data to the internal storage of the smartphone and convert it into the standard protocol format of the application. Depending on the RPi and the sensor configuration, the app allows for the use of several sensors simultaneously. The analysis tool of the application adapts data of external measurements to obtain further results immediately.

## 3. System Performance

### 3.1. Sampling Regularity and Time Synchronization

A test was conducted to assess the quality of the presented measurement system. One of the fundamental characteristics of the measurement system to be used for dynamic measurements is the regularity of sampling. It is critical for the system to achieve a constant sampling rate, i.e., the sensors need to be sampled at uniformly distributed time instances to ensure the integrity and quality of the measurement process.

**Figure 6:**
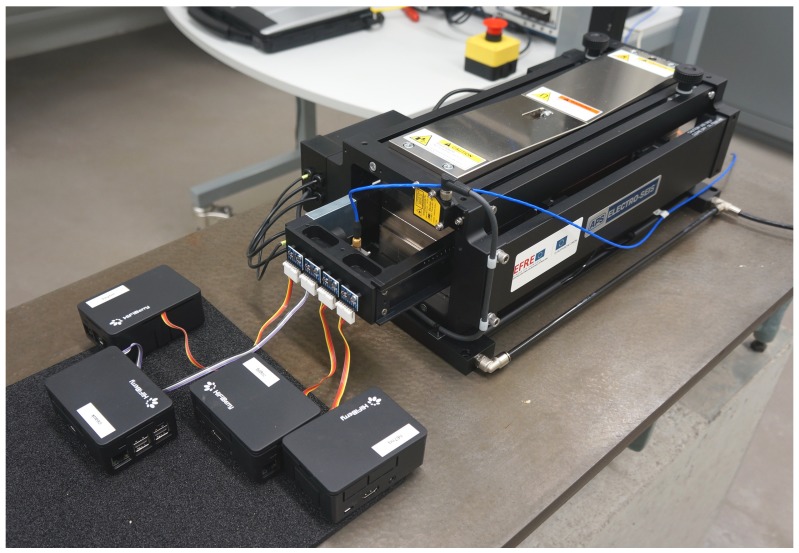
Measurement setup with accelerometers mounted to the moveable piston of the long stroke vibration exciter. MEMS-based accelerometer connected to RPi A, B, C, and D are attached at the front side of the piston; capacitive accelerometer mounted on the back side.

The test was performed by mounting four MPU-6050 accelerometers on a calibration shaker (APS 113 ELECTRO-SEIS) as shown in Figure 6. The ±2 g MEMS-based accelerometers were attached to the front side of the moveable piston of the shaker. The shaker is equipped with a ±3 g highly accurate capacitive accelerometer (PCB 3701G2FA3G) [27], used to control the piston movement. The capacitive accelerometer is mounted to the back side of the piston and therefore measures acceleration at the same moving shaker element as the four MEMS accelerometers, which were each connected to an I${}^{2}$C port of an individual RPi.

**Figure 7:**
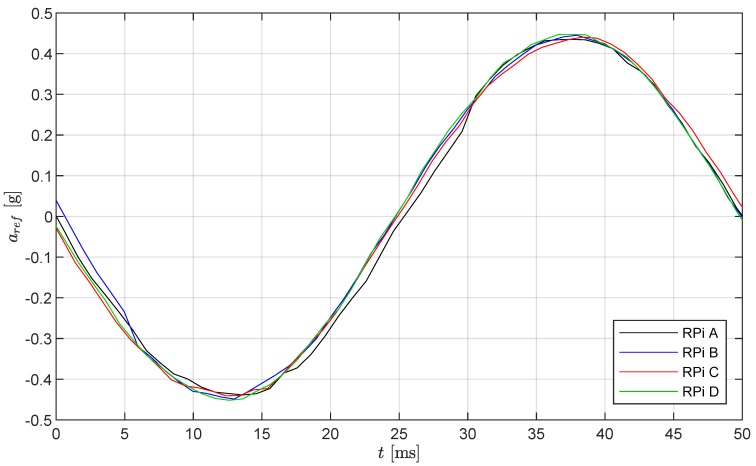
Sample time history of one cycle of the measured accelerations from MPU-6050 accelerometers connected to four separate RPis with a sampling frequency of 1 kHz and a period of *T* = 50 ms.

The shaker was set to vibrate harmonically with a frequency of 20 Hz and with a peak amplitude of 0.45 g. The measurements were taken for 60 min with a sampling frequency of 1 kHz. Since the goal of the test is the investigation of the regularity of sampling and the time synchronization between the RPis, only the measured time histories of the RPi measurement systems are analyzed here. A sample time history window of the sensor measurements for one cycle of oscillation is presented in Figure 7. Note that the amplitude differences visible between the four sensors subjected to the same physical motion is mostly due to the appreciable noise of the low-cost MEMS sensors and not related to the quality of the RPi systems.

**Figure 8:**
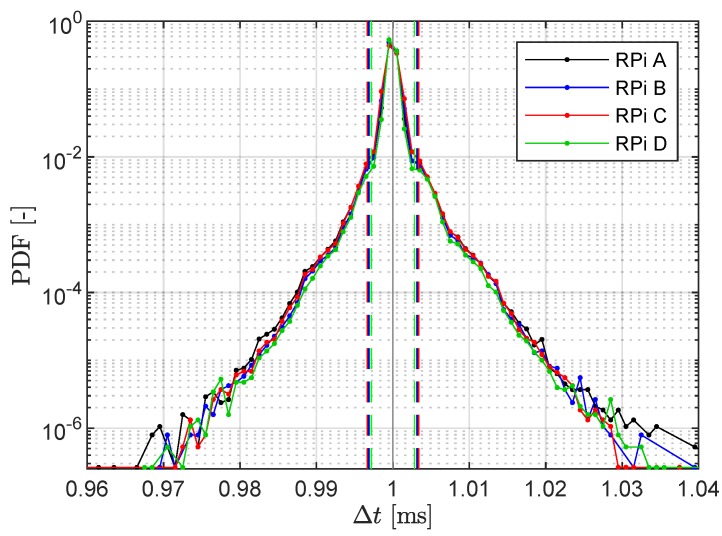
Normalized histograms of measured time increments Δt from MPU-6050 accelerometers with a sampling frequency of 1 kHz, i.e., nominal Δt = 1 ms. (Dash lines indicate the corresponding 99th percentile.)

First, the regularity of the sampling was examined. For each RPi, the distributions of the measured time steps Δt were determined. Setting a sampling rate of 1 kHz, the sampling period Δt nominally is 1 ms. The histograms, given in Figure 8, show the distribution of the time steps as measured. The four systems show similar sampling behavior with a time step closely varying around the nominal value of 1 ms, and the difference to that nominal values not exceeding 60 μs. The dashed lines indicate the 99th percentile and lie within 4 μs of the nominal value, confirming robust regularity. Further sampling quality measures are presented in Table 2. Ninety-nine percent of the time steps differ by less than 3.4 μs, the maximum difference to the ideal sampling step being less than 60 μs.

**Figure 9:**
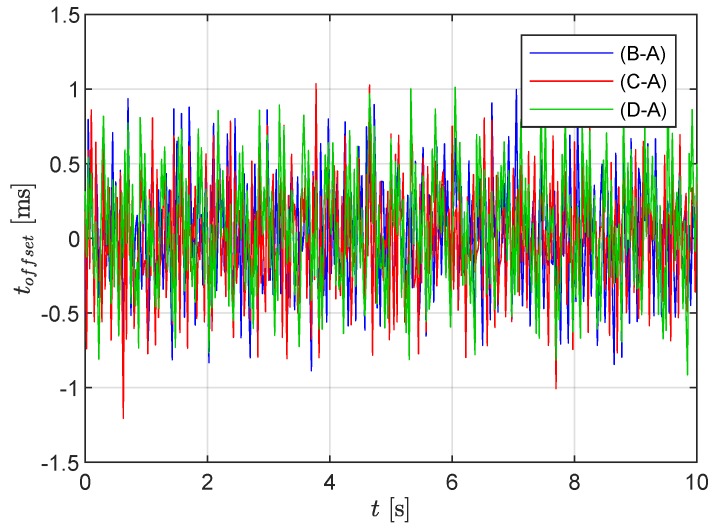
Determined time history of the offset $t_{offset}$ over a sample time window of 10 s.

The accuracy of the time synchronization between the RPis was also verified for the measurement system. Thereby, RPi A was considered as a reference, and the time offsets of the other three signals were computed. For each of the signals, the intersections with the abscissa, i.e., the time instances of zero acceleration, were determined. The offset $t_{offset}$ was then determined from the difference of the intersection times from the signals (RPi B, C, and D) to the reference. Figure 9 shows the time-varying offset for RPi B, C, and D for a sample 10 s window relative to the reference measurement. It can be observed that the measured offset shows a noisy behavior and varies mostly within the range of ±1 ms with maximum absolute offset of 1.2 ms. In Figure 7, it can be seen that time variations of the order of the time step occur, leading to randomized time shifts between the signals of the same order as shown in Figure 9. In other words, an uncertainty of relative timing between signals occurs but does not significantly exceed the length of one sampling time step. The reader is reminded that the MPU-6050 accelerometer is low-cost and that sensor communication leads to randomized delays.

**Figure 10:**
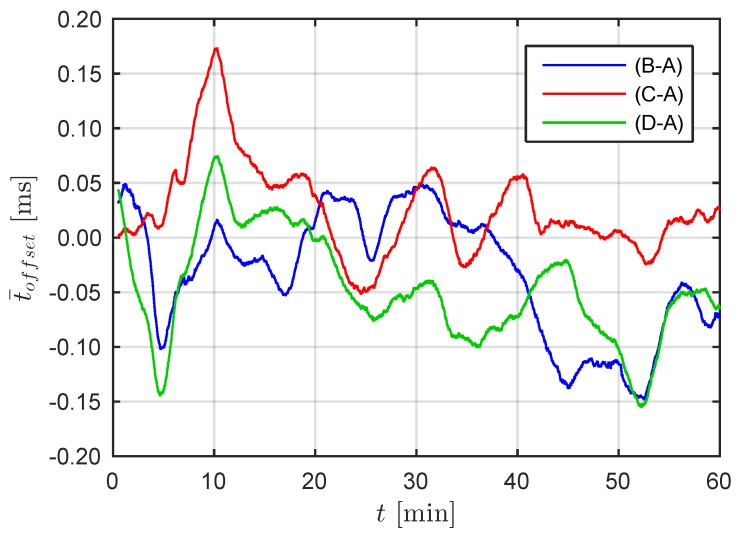
Moving average of the time offset ${\bar{t}}_{offset}$ over 60 min of the RPi B–D measurements compared to reference RPi A (window size 60 s).

The determined time offsets were further processed to obtain a mean offset over the full measurement duration. Thereby, a moving average with a window size of 60 s was computed, thus eliminating the randomized sampling discussed above and leading to a measurement of clock synchronization of the units. The time histories of the mean offset ${\bar{t}}_{offset}$ from RPi B to D with respect to RPi A are displayed in Figure 10. The variations over 60 min are less than ±0.18 ms and therefore indicate very good time synchronization. In general, the study presented here confirms an effective time synchronization of the implementation that is within the range of what is required in typical SHM applications.

Additionally, several test measurements were conducted with different sampling rates over a time span of an hour. The system was put under a heavy CPU load by spawning four processes generating random numbers. This scenario was chosen to verify that the real time prioritized polling process is not hampered by competing tasks running on the nodes. Table 3 summarizes the maximum and minimum values of time increments from the measurement for all sensors as well as the time increment and the 99% confidence interval for the recorded time series. It can be observed that the 99% confidence interval is less than 0.025 ms for all measurements. Overall, the results indicate a stability in the sensor sampling that is sufficient for most SHM applications and is comparable to the performance of dedicated microcontrollers.

### 3.2. Limitations of the System

The main limitations of a smartphone as a measurement node are the on-board components such as accelerometer, processor and storage space, all of which cannot normally be exchanged. If these components are not sufficient for a measurement task, a different smartphone needs to be used, if available. As previously described, the quality of vibration measurements primarily depends on the sampling rate and resolution of the accelerometer used. Usually the sampling rate of smartphones is in the range of 100–250 Hz, which may not be sufficient for precise measurements on high frequency building elements but still covers a sizeable proportion of use cases in SHM. By using the Android API to query the capabilities of the on-board accelerometer sensor characteristics are reported that are considerably lower than those stated in corresponding data sheets of the embedded sensor hardware. A detailed review of accelerometer properties in several smartphone models and actual test measurements can be found in [6].

The current implementation of the IP address assignment scheme bears the risk of potential address collisions. Configuration of the mesh interface is tightly coupled to the hardware platform and needs to be redesigned for deployment on single-board computers with different WiFi adapters. These shortcomings can be addressed by switching to an IPv6 address scheme. Additionally, the implementation of the OLSR protocol in version 2 affords device independent generation of IPv4 addresses by using the IPv6 link-local address of the local interface as a seed and verifying that the generated address does not collide with any known nodes in the one- and two-hop neighborhood in the network [28].

## 4. Applications

The technology platform as it is presented in this paper has been used for several measurement campaigns including the following monitoring tasks:

- the identification of natural frequencies of cantilevered balcony plates using a meshed sensor network controlled by the smartphone application,
- the identification of vertical and rotational natural frequencies of a dynamic wind tunnel rig using RPi and external sensors,
- the identification of natural frequencies of a pole structure for model updating,
- the determination of prestressing forces of external tendons in a highway bridge [15],
- the identification of stay cable forces at Queensferry Crossing bridge,
- the determination of peak velocities from acceleration measurements at a bridge pier during blast operation, and
- the identification of modal properties of a simply supported steel beam.

In most of these measurement tasks, the natural frequencies needed to be identified. Using a high-quality reference measurement system in addition to the presented mobile measurement system, very good agreement in the identified natural frequencies was found, highlighting the accuracy and validity of vibration measurements taken by the mobile measurement system.

It should be noted that the presented measurement system was mostly applied for short-term vibration measurements. Utilizing a standard rechargeable power bank battery with a capacity of 10.000 mAh, in-situ measurements of up to 20 h can be performed. Continuous longer term measurements would require an extended power supply, and in the current hardware configuration the measurement system is neither water-proof nor dust-proof. However, RPi and sensor hardware can be integrated in different enclosures to ensure a certain ingress protection (IP) grade depending on the operational conditions. Attachment to the structure can then be faciliated by a bolted connection. In a previous study, a system composed of RPi and highly accurate MEMS-based inclination sensors, integrated in a polycarbonate enclosure, was employed to monitor a long span roof beam of an industrial plant subjected to snow load [29,30]. During winter, the inclination was measured continuously, and the height of the snow layer was determined from measured data. A wired connection was used since power and network access was available. The system was configured to store large amounts of measurement data on external USB storage media. Additionally, the obtained data were periodically synchronized with a remote file server, and system health information, such as CPU temperature and load, was sent to a central server.

The focus of this paper, however, is on short-term vibration measurements taken by the proposed wireless sensor network. In order to show its practicality and to study the performance during field tests, the last three monitoring tasks of the above presented list will be discussed here in detail.

### 4.1. Estimation of Stay Cable Forces at Queensferry Crossing

During the construction of Queensferry Crossing (QFC), a cable-stayed bridge over the Firth of Forth near Edinburgh, vibration measurements were taken on stay cables to determine their tension forces. The cable forces were also available directly from stressing operations during the installation of the cables, thus serving as a reference for the results determined by smartphones as well as the RPi-based measurement system. A brief summary of the investigations will be given here. For further details, the reader is referred to [6].

In a preliminary test, the accelerations were recorded under different manual excitations at selected stay cables. The extracted frequency spectra revealed that several modes could be identified, while the symmetric modes of vibration were more prominent. The fundamental frequency was not found due to the nature of the manual excitation near the lower cable anchorage. The frequencies that could be identified with the smartphone and the RPi measurement system agreed well with differences for higher modes typically being less than 1%. It should be noted that, even under small acceleration amplitudes, frequencies could be identified.

In a second test, the influence of the excitation was investigated with manual excitations compared to ambient vibrations that were mainly induced by wind. Acceleration measurements were taken at different stay cables by two different smartphones. The natural frequencies were extracted, and the cable force was determined by employing a non-linear force-frequency relationship that was obtained by numerical simulations. The comparison to the reference force showed that calculated forces differed by no more than 1% and were on average within 0.5% of the references, which was within the expected accuracy of the reference forces.

Further vibration measurements were conducted at several other stay cables under ambient excitation. Thereby, the external sensor MPU-6050 was connected to the RPi, and the smartphone was used as a central control unit. This setup was chosen because it features both sufficiently high resolution and a sampling rate necessary for the validation under ambient vibrations. The measurements showed that at least 10 natural frequencies could be identified in each of the measurements for the investigated stay cables. Again, the cable forces were identified applying the non-linear force-frequency relationship, and a good agreement with the reference cable forces was found. The predicted cable forces were within the limit of a 1.5% maximum and less the 1% on average. To sum up, the RPi-based system and a specific smartphone (Sony Xperia Z5) were able to identify frequencies with sufficient accuracy even under ambient wind excitations.

### 4.2. Determination of Peak Velocities during Blast Operation at Schindgraben Bridge

**Figure 11:**
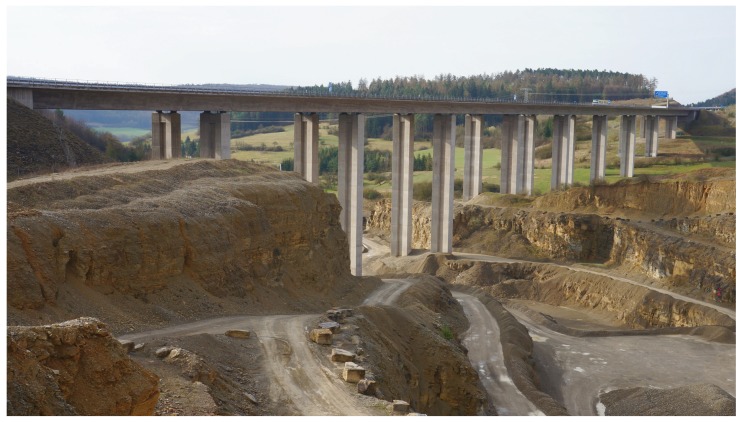
Highway bridge partly founded in a mining site.

The mobile measurement system was further employed in a different usage scenario at a highway bridge located in Central Germany. The considered box girder bridge has unique site conditions being partly founded in a quarry (see Figure 11) where every few months explosive excavations are performed. The monitoring task was to determine the maximum vibration velocity at the top of the tallest bridge pier due to ground shaking induced by these blasts. An allowable peak velocity had been previously determined from detailed studies of the structural performance under typical base excitation, thus serving as a threshold value to judge structural integrity on the basis of on-site measurements.

The acceleration at the top of the tallest bridge pier was measured in three axes by an external MPU-6050 sensor connected to the RPi-based measurements system, two smartphones (Sony Xperia Z5, Nexus 4), and a tablet (Nexus 7) as well as a high-quality reference system. The recorded acceleration time histories were then integrated over time to compute the velocity time history and to determine the absolute peak velocity. Thereby, signal processing such as filtering and detrending of the individual signals was necessary. The peak velocities determined by the different systems are compared in Table 4. In comparison to a high-quality reference sensor system, the results obtained by the alternative mobile measurement systems are generally in good agreement. The superiority of the RPi linked to the MPU-6050 accelerometer over the smartphones in terms of sampling rate can be seen in the smallest deviations which are below 1.2% for all the axes, while the peak velocities found of the other systems are within the range of 3–9% and show notable variations between the axes.

It should be noted that, shortly before the blast, the bridge had to be closed for traffic. The reopening of the bridge required the explicit approval of an engineer whose decision is based on the non-exceedance of the peak velocity threshold. In order to keep the interruption of the traffic on the highway as short as possible, it is crucial to ensure reliable, robust, and fast data processing. Since all the necessary data processing steps are implemented in the smartphone app, measurement and data analysis is integrated in a single mobile device, and the obtained results show satisfactory accuracy for judging the acceptability of the measured vibrations. The system employing a smartphone as a control unit for the RPi-based measurement system with MPU-6050 is a powerful solution for the engineer in charge. Knowing that the obtained peak velocities are typically well below the threshold value, it is also possible to employ the smartphone Nexus 4 as a standalone solution by defining the limit of accuracy. Since there is a trend of increasing sampling rates with which smartphones can record accelerations, it is expected that results of higher accuracy will be found with future phones, making smartphones an even more attractive choice.

Since the smartphone is highly mobile and the smartphone application can trigger and control measurements, a second smartphone was attached at the bottom of the pier without much effort to collect additional information about the structural behavior. The presented measurement system also allows simultaneous measurement at all bridge piers while the RPi-based wireless sensor network is employed. RPi nodes can also be used as a WiFi-extender in the case of long distances, and even smartphones can easily be integrated into the network.

The determination of natural frequencies and integral values such as velocities and displacements can be obtained from a single sensor attached to the structure at a suitable position. The benefit of having a mesh-based wireless sensor network providing synchronized sensor nodes is that dynamic properties such as mode shapes and the corresponding structural damping can also be identified. This advantage will be demonstrated in the following sample application.

### 4.3. Identification of Modal Information of a Steel Beam

**Figure 12:**
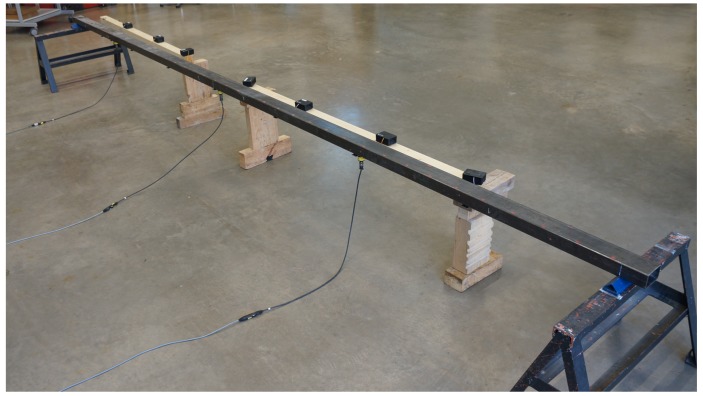
Experimental setup with sensors attached at the bottom of the beam. (Wooden pieces are used as support for the sensor nodes.).

This application considers a simply supported steel beam as shown in Figure 12. The beam has a hollow profile of dimensions 100 × 60 × 3 mm and spans 5.80 m. The goal was to determine the frequency, the shape, and the damping of the first five vertical bending modes as in typical SHM tasks.

**Figure 13:**
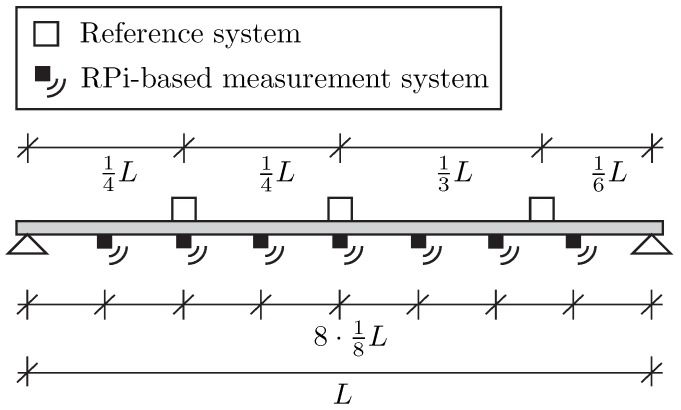
Schematic of the sensor setup at the steel beam.

The beam structure was excited by regular hammering at distinct locations (∼0.3·L) to excite multiple modes of the beam. Two different measurement systems are available for the measurement campaign: first, a high-quality reference system including highly sensitive piezoelectric sensors (PCB 393A03) connected to a data acquisition unit featuring a synchronous input channel and 24 bit signal conversion; second, the alternative RPi-based measurement system using external MPU-6050 sensors [31]. In total, three piezoelectric sensors and seven sensor nodes were employed in the vibration test. The sensor setup is shown in Figure 12 and Figure 13. The reference sensors are placed at midspan, at 1/4 and 1/6 of the span, where maximum displacement of the first, second and third mode is expected. The seven wireless sensor nodes are placed equally throughout the length of the beam with a center-to-center distance of 0.725 m. The sensing characteristics and the costs of the two applied measurement systems are summarized in Table 5. The superiority of the reference system can be seen from the much higher resolution. In addition to the synchronous data acquisition, it features an integrated anti-aliasing filter and allows for sampling rates up 40 kHz. However, the sampling rate was set to 1024 Hz for this experiment.

**Figure 14:**
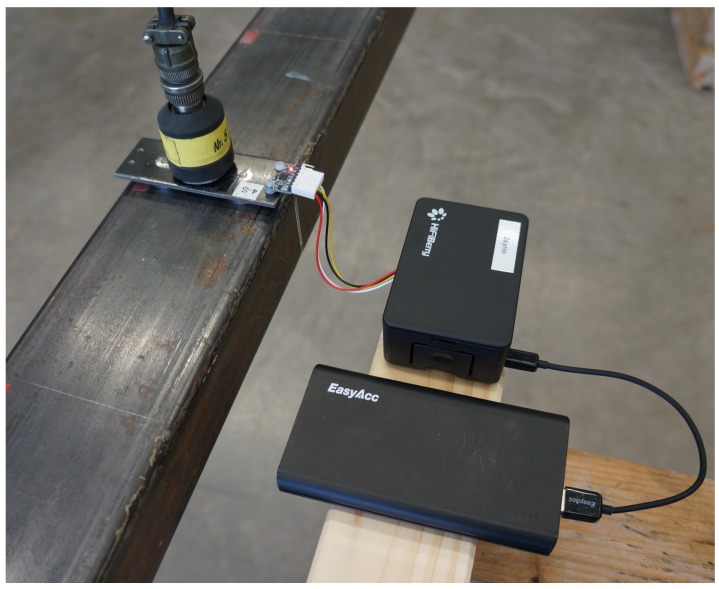
View of the sensor mounting plate equipped with a high-quality piezoelectric sensor and an external accelerometer attached to the RPi.

Both piezoelectric and MEMS accelerometers were mounted to steel plates that were attached to the beam by means of two magnets, as shown in Figure 14. The RPi sensor nodes were located next to the beam on wooden supports. After powering the RPi, the wireless network and the meshing was set up automatically. Prior to the measurements, the time synchronization of the sensor nodes was conducted via the previously presented frontend software, which was further used to control the measurements.

The accelerations were recorded for more than 100 s in order to achieve a frequency resolution higher than 0.01 Hz. The time histories were measured simultaneously by all sensors of both systems.

For further processing, the acceleration time histories of the MEMS accelerometer were resampled to a frequency of 2000 Hz. System identification was performed using the Matlab toolbox MACEC [32], which employs the covariance-driven approach of stochastic subspace identification and thus determines the modal information of the beam from the recorded sensor data. Suitable modes were then chosen from the stabilization diagram. The natural frequencies and damping ratios obtained from the two applied systems are compared in Table 6. The natural bending frequencies have been identified and show good agreement between the two systems with a maximum difference of 0.03 Hz in the second mode. Regarding the determined damping, a greater deviation that is more dominant for the lowest modes can be seen. It should be noted that the considered steel beam has a very low inherent damping, making it difficult to measure accurately.

**Figure 15:**
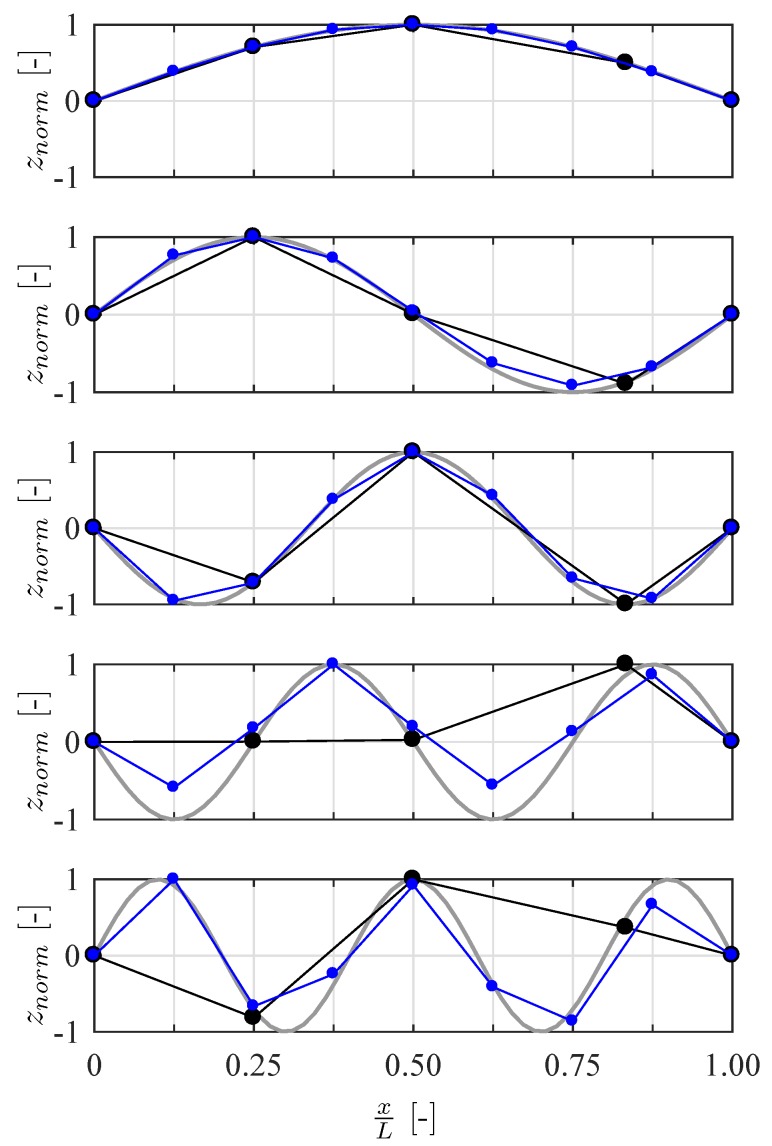
Comparison of the identified mode shapes obtained by reference system (―●―) and RPi-based measurement system (―●―). The first five mode shapes are displayed along with the theoretical modes shapes (━).

The mode shapes obtained for the first five modes are shown in Figure 15 alongside those computed analytically. Note that the analytical mode shapes are only an approximation of the real behavior, as they disregard sensor masses as well as potential imperfections in the system due to variations of weight and stiffness. Both systems show good agreement especially in the first three modes. The general shape of the fourth and fifth mode are still determined well from RPi measurements; however, the amplitude at the sensor locations shows deviations from the analytical shape. The reference measurement system shows agreement with the analytical shape that is broadly similar to that of the RPi system. Still, the fourth mode shows a significantly better performance, as indicated by the modal assurance criterion (MAC) given in Table 6. The MAC value for the highest modes is still greater than 0.98 for the reference system, while it drops to 0.91 for the modes identified from synchronous RPi measurements. However, the reference mode shapes lack supporting points to properly reflect the higher sine waves due to the smaller number of sensors used. It is worth considering this aspect in the context of the cost of the measurement system. The results clearly show that the RPi system obtains modal information, specifically mode shapes, not available from a reference system of at least 20 times the cost. Of course there are SHM applications where such a highly accurate system may be strictly required. Furthermore, it is possible to overcome the above-highlighted shortcomings of a limited sensor count by performing multiple measurements at different sensor arrangements. Yet, where the presented WSN solution is sufficiently accurate for the task at hand, it represents an attractive alternative to traditional technologies, including proprietary WSN systems, at a fraction of their cost.

## 5. Conclusions

This paper has presented a combined hardware and software solution capable of performing highly accurate measurements in a WiFi-based meshed configuration of sensing nodes. The main focus was the coherent software framework developed for off-the-shelf and cost-effective microcomputer hardware such as RPi, turning them into sensing nodes that also perform network communication. A smartphone app that facilitates the initiation and management of the measurement process and allows for data processing and storage is presented. Further, the current implementation, by polling their internal sensors, allows for measurement with smartphone devices integrated into the mesh. Several test measurements that focus first on the specific data acquisition characteristics of the systems in a meshed configuration are reported. The results confirm the high performance of the measurement system in terms of stable sampling at high sampling rates up to 1 kHz and an accurate time synchronization between nodes, with time shifts being reliably under 0.2 ms. A second set of tests showed that the systems can perform well in typical SHM tasks such as the frequency measurement and force computation of stay cables, velocity measurement based on acceleration sensing, and modal identification of structures. Limitations seen in the results provided are mostly due to the use of very cheap MEMS-based sensors, where the costs of a single node are below 50 €, not accounting for power supply and enclosure.

In addition to the quality of measurements, workflows are relatively simple and user-friendly due to the highly integrated nature of hardware and software components. The RPi-based monitoring system presented is a flexible, highly mobile, cost-effective, and robust system that has been found to offer measurement characteristics in a meshed configuration that is sufficient for a wide range of SHM applications and that are superior to many standard WSN systems. Finally, the solution presented here is not based on any proprietary technology and lends itself well to teaching purposes.

## Figures and Tables

**Table 1:** Summary of implemented sensor driver modules.

Product Name	Product Type	Vendor	Sensor Type	Hardware Interface
MPU-6050	Sensor	InvenSense	MEMS accelerometer	I${}^{2}$C
MPU-9250	Sensor	InvenSense	MEMS accelerometer	SPI
ADS1115	ADC	Texas Instruments	Any analog sensor	I${}^{2}$C
CXL02TG03	Sensor	Memsic Inc.	MEMS accelerometer	I${}^{2}$C (ADS1115)
ADXL335	Sensor	Analog Devices	MEMS accelerometer	I${}^{2}$C (ADS1115)
SI-11.S1.C-30	Sensor	First Sensor	Inclinometer	SPI
HX711	ADC	Avia SC	Strain gauge amplifier	Serial

**Table 2:** Summary of differences of measured time steps Δt from MPU-6050 accelerometers to the nominal value $\Delta t_{nom}$ with a sampling frequency of 1 kHz.

Sensor	$\Delta t_{99\%}-\Delta t_{\mathrm{nom}}$	$\Delta t_{\max}-\Delta t_{\mathrm{nom}}$
	[ms]	[ms]
A	0.0032	0.045
B	0.0031	0.040
C	0.0034	0.058
D	0.0028	0.041

**Table 3:** Summary of differences of recorded time increments Δt and corresponding maximum offsets to the nominal value $\Delta t_{nom}$ for different sampling frequencies for a measurement polling an MPU-6050 accelerometer.

Sampling Rate	$\Delta t_{\mathrm{nom}}$	$\Delta t_{99\%}-\Delta t_{\mathrm{nom}}$	$\frac{\Delta t_{99\%}-\Delta t_{\mathrm{nom}}}{\Delta t_{\mathrm{nom}}}$	$\Delta t_{\max}-\Delta t_{\mathrm{nom}}$	$\frac{\Delta t_{\max}-\Delta t_{\mathrm{nom}}}{\Delta t_{\mathrm{nom}}}$
[Hz]	[ms]	[ms]	[%]	[ms]	[%]
50	20	0.0201	0.10	0.0534	0.27
100	10	0.0136	0.14	0.0434	0.43
200	5	0.0125	0.25	0.0325	0.65
500	2	0.0068	0.34	0.0373	1.86
750	1.33	0.0056	0.42	0.0454	3.41
1000	1	0.0037	0.37	0.0331	3.31

**Table 4:** Comparison of the different measurement systems in terms of their sensing characteristics and determined peak velocities along with the relative difference to the high-quality reference system.

System	Sampling Rate	Resolution	Peak Velocity [mm/s]			Relative Difference [%]		
	[Hz]	[bit]	x-axis	y-axis	z-axis	x-axis	y-axis	z-axis
Reference system	2400	23	5.63	6.22	5.32	-	-	-
RPi and MPU-6050	565	14	5.60	6.28	5.38	−0.53	+0.96	+1.13
Nexus 4	177	15	5.34	6.05	5.09	−5.15	+2.73	+4.32
Sony Xperia Z5	177	14	5.31	6.43	4.87	−5.58	+3.38	+8.46
Nexus 7	219	13	5.66	5.66	5.28	+6.75	−9.00	−0.75

**Table 5:** Properties of the utilized measurement systems for the steel beam experiment [31].

System	Sampling Rate	Resolution	Noise Density	Cost
	[Hz]	[bit]	[μg/$\sqrt{\mathrm{Hz}}]$	[€]
Reference system	1024	23	1	12,000
RPi and MPU-6050	1000	15	400	<500

**Table 6:** Comparison of the identified dynamic properties of the steel beam, where δ is the relative difference δ between the reference and the RPi-based measurement system.

Mode	Natural Frequency			Critial Damping Ratio			MAC		
	Ref. [Hz]	RPi [Hz]	δ [%]	Ref. [%]	RPi [%]	δ [%]	Ref. [-]	RPi [-]	δ [%]
1	5.857	5.859	0.22	0.032	0.023	−28.8	1.000	1.000	0.0
2	22.286	22.252	−1.51	0.243	0.150	−38.3	1.000	0.996	0.4
3	50.601	50.611	0.21	0.078	0.078	−0.7	1.000	0.998	0.1
4	91.361	91.362	0.01	0.262	0.233	−11.0	0.999	0.910	8.9
5	131.330	131.350	0.16	0.144	0.133	−7.7	0.984	0.969	1.4

## Abbreviations

The following abbreviations are used in this manuscript:

ADC	Analog-to-Digital Converter
AP	Access Point
API	Application Programming Interface
DHCP	Dynamic Host Configuration Protocol
FFT	Fast Fourier Transform
GPIO	General Purpose Input Output
HNA	Host and Network Association
HTTP	Hypertext Transfer Protocol
IP	Ingress Protection
IP	Internet Protocol
I${}^{2}$C	Inter-Integrated Circuit
MAC	Media Access Control
MAC	Modal Assurance Criterion
mDNS	Multicast Domain Name System
MEMS	Micro-Electro-Mechanical Systems
NTP	Network Time Protocol
OLSR	Open Link State Routing
PZT	Piezoelectric lead Zirconate Titanate
QFC	Queensferry Crossing
RPC	Remote Procedure Call
RPi	Raspberry Pi
SHM	Structural Health Monitoring
SPI	Serial Peripheral Interface
